# Long-term outcomes after enterostomy for very early-onset inflammatory bowel disease with interleukin-10 signaling deficiency

**DOI:** 10.1186/s12876-023-03051-4

**Published:** 2023-11-20

**Authors:** Zifei Tang, Song Sun, Min Ji, Peng Shi, Yuhuan Wang, Zhiheng Huang, Ying Huang

**Affiliations:** 1 Department of Gastroenterology, Children’s Hospital of Fudan University, 399 Wanyuan Road, Shanghai, Minhang District, 201102 China; 2 Department of Surgery, Children’s Hospital of Fudan University, Shanghai, 201102 China; 3 Department of Radiology, Children′s Hospital of Fudan University, 201102 Shanghai, China; 4 Pediatric Clinical Research Unit, Children’s Hospital of Fudan University, Shanghai, 201102 China

**Keywords:** Very early-onset inflammatory bowel Disease, interleukin-10 receptor gene, Enterostomy, Stoma closure, Hematopoietic stem cell transplantation

## Abstract

**Background:**

Very early-onset inflammatory bowel disease (VEOIBD) with interleukin-10 (IL10R) signaling deficiency usually requires enterostomy in patients who are refractory to traditional treatment. This study aimed to evaluate long-term outcomes after enterostomy for VEOIBD patients with IL10R signaling deficiency.

**Methods:**

The medical records of all patients undergoing enterostomy for signaling deficiency were retrospectively assessed during 2012.1–2022.7 in a tertiary teaching hospital, Children’s Hospital of Fudan University, Shanghai, China. Data on disease history, diagnosis and details of enterostomy and stoma closure and follow-up were collected. Univariate and multivariate logistic regression analyses were used to evaluate the risk factors associated with the long-term outcome of delayed stoma closure.

**Results:**

A total of 46 patients underwent an enterostomy, 19 who required emergency enterostomy and 27 with selective enterostomy. After ten years of follow-up, 35 patients underwent hematopoietic stem cell transplantation (HSCT), and 25 patients were alive after HSCT. The median timeframe between HSCT and stoma closure was 19.6 [15.9,26.2] months. Nineteen patients underwent stoma closure and had an average age of 3.9 ± 1.5 years; 6 patients were waiting for stoma closure. Based on a univariate logistic model, risk factors significantly associated with late stoma closure were age at enterostomy and age at HSCT. However, multivariate logistic regression showed no statistically significant factor associated with late stoma closure. There was no significant difference between the stoma closure group and delay closure group in the z scores of weight for age at follow up.

**Conclusions:**

This study determined the long-term outcomes after enterostomy for VEOIBD with interleukin-10 signaling deficiency. The appropriate time point of enterostomy and HSCT may improve quality of life in the long term.

## Introduction

Very early-onset inflammatory bowel disease (VEOIBD), caused by defects in interleukin-10 (IL-10) signaling, including defects in IL10, IL10RA, and IL10RB, is an autosomal recessive disorder [1, 2]. This type of VEOIBD often presents as diarrhea, perianal diseases, oral ulcers, intestinal ulcers, perforation, and obstruction [3, 4]. VEOIBD patients are refractory to conventional therapies and require early surgical intervention and further hematopoietic stem cell transplantation (HSCT) [5, 6].

In our previously reported study, the complications of enterostomy and related risk factor analysis of VEOIBD with IL-10 signaling deficiency in our IBD center were determined [7]. However, the long-term outcomes of these patients after enterostomy are still unclear, and some risk factors for stoma closure are especially unclear. This study aims to collect the data of these patients after enterostomy, report their long-term outcomes and shed light on higher quality management for VEOIBD patients with IL-10 signaling deficiency after enterostomy.

## Methods

This study was approved by the Ethics Committee of the Children’s Hospital of Fudan University. The medical records of all children undergoing enterostomy for VEOIBD with IL-10 signaling deficiency from 2012.1 to 2022.7 in the Children’s Hospital of Fudan University were retrospectively assessed. Data on disease history, genetics, enterostomy, HSCT and stoma closure were reviewed retrospectively; the research methods and most of the patients were reported in our previous study [8], however more details are described in this study. For the included patients, the available height, weight, weight-for-age (WFA) Z score, height-for-age (HFA) Z score, and BMI Z score were determined using the World Health Organization (WHO) Anthro software (version 3.2.2) [4].

The decision to perform stoma closure primarily involved three factors. First, the intestinal lesions were much improved after colonoscopy examination. Second, immune reconstitution was successful after HSCT. Last, the severe enterostenosis or and perianal lesions were staged treated surgically before stoma closure.

Data were analyzed using SPSS 24.0 for Windows (SPSS Inc., Chicago, IL). Continuous data are presented as the mean and SD or median and interquartile range. Risk factors were analyzed with logistic regression. Because the traditional p level of 0.05 might fail to identify variables known to be important, we chose only factors that had *P* values < 0.1 in univariate analysis for inclusion and in multivariate analysis.

## Results

### Baseline characteristics

**Fig. 1:**
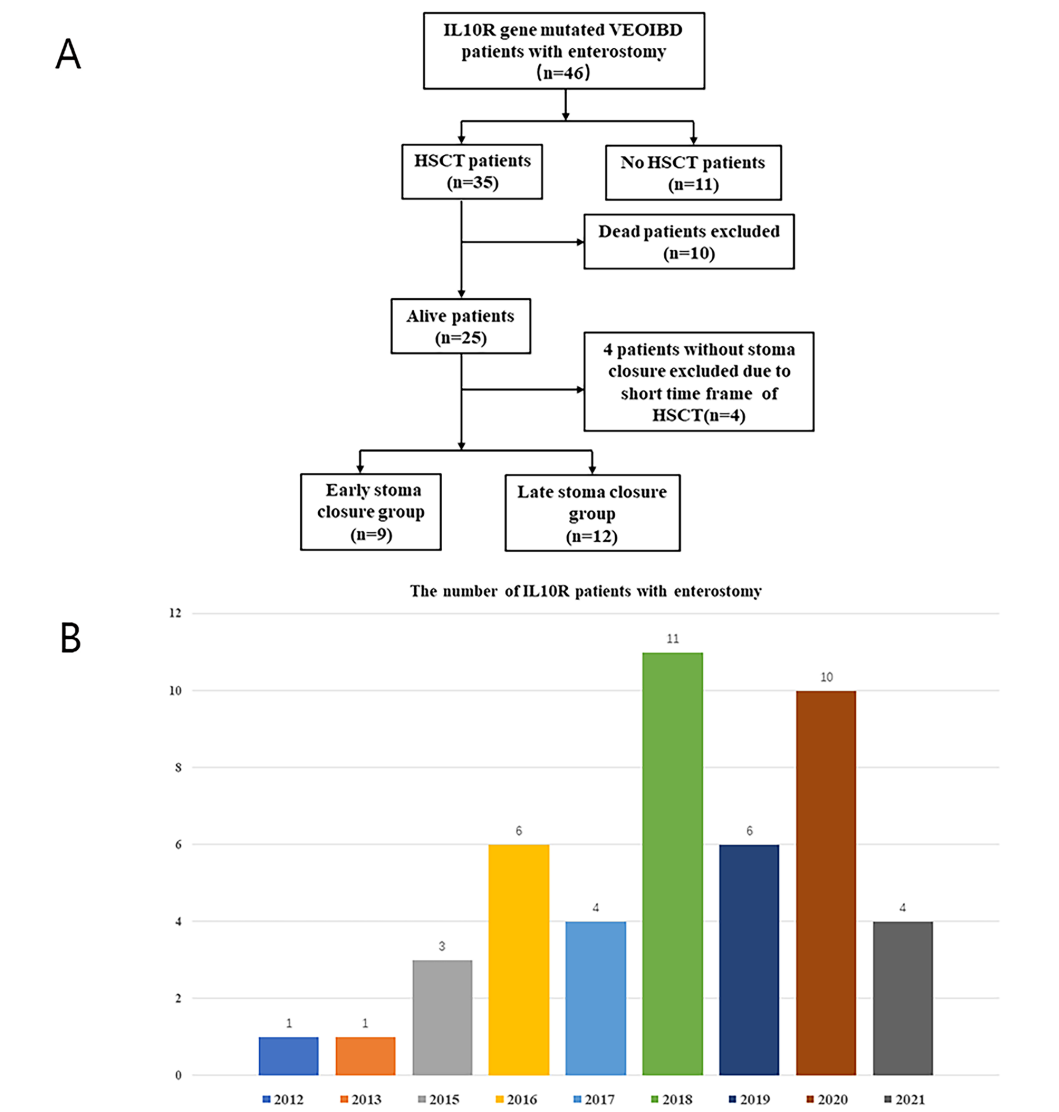
**A:** Flow chart of the inclusion and exclusion of the patients based on the diseases diagnosed in this study. **B:** The number of VEOIBD patients with IL10R gene mutations and enterostomy from 2012 to 2021

A total of 133 VEOIBD patients with IL-10 signaling deficiency were enrolled in our single IBD center from 2012.1 to 2022.7. Among them, the clinical data of 46 patients with enterostomy were further collected (Fig. 1A). The demographic features of the patients are summarized in Table 1. Within 10 years of diagnosis, 34.6% of the incident patients had an enterostomy (46/133). Among them, 21 patients were male, and 25 patients were female. The age at diagnosis was 12.7 [5.7, 24.2] months. There were 37 compound heterozygous mutations of the IL10RA gene, 8 homozygous mutations of the IL10RA gene and 1 homozygous mutation of the IL10RB gene. A total of 76.1% (35/46) of the patients underwent HSCT, and 11 patients did not undergo HSCT for various reasons.

**Table 1:** The demographic features of the VEOIBD patients with IL10R gene-mutations and enterostomy

Character	
Number of patients	46
Male: Female (n)	21:25
Age at diagnosis (m)	12.7[5.7,24.2]
Duration of follow-up (m)	21.2[9.6,31.7]
Fever (n)	43
Diarrhea (n)	43
Oral ulcer (n)	29
Perianal disease (n)	42
HSCT: none HSCT(n)	35:11
Surgery (Emergency: selective) (n)	19:27
Alive: dead(n)	31:15
Alive: HSCT: none HSCT(n)	25:6
Dead: HSCT: none HSCT(n)	10:5

### Enterostomy in VEOIBD patients with IL10R gene mutations

The number of VEOIBD patients with IL10R gene mutations and enterostomy from 2012 to 2021 are shown in Fig. 1B. The quartile age of enterostomy for VEOIBD patients with IL10R gene mutations was 11.5 [5.7, 23.3] months, and it was 11.5 [5.6, 36, 6] months for male patients and 7.9 [5.7, 23.1] months for female patients. There was no significant difference between the two sexes (*P* = 0.48).

The patients with enterostomy were divided into an emergency enterostomy group and a selective enterostomy group. The reasons for emergency enterostomy and selective enterostomy are provided in Table 2. There was a significant difference between the ages of the two groups (7.9 [4.3, 26.0] vs. 12.9 [7.4, 23.4] months, *P* = 0.09). Among them, 31 patients were still alive, and 15 patients had died. Among the surviving patients, 25 underwent HSCT. Among the 15 deceased patients, 11 underwent emergency enterostomy (6 underwent HSCT), and 4 underwent selective enterostomy (4 underwent HSCT). We found that the patients in the emergency enterostomy group had higher mortality than those in the selective enterostomy group (11/19 vs. 4/23, *P* = 0.001).

**Table 2:** Reasons for emergency enterostomy or selective enterostomy

Reason of enterostomy	Number(%)
**Emergency enterostomy** (n)	19 (41.3)
Intestinal perforation	11(23.9)
Intestinal obstruction	8 (17.4 )
Intestinal severe stenosis	4 (8.7)
**Selective enterostomy**	27 (58.7)
Aggressive infection/inflammation	2 (4.3)
Severe perianal lesions	11 (23.9)
Severe intestinal lesions	12 (26.1
Intestinal stenosis	4 (8.7)
**Type of stoma**	
ileal end stoma	39 (84.8)
ileal double barrel stoma	3 (6.5)
colon end stoma	4 (8.7)

### Stoma closure

**Fig. 2:**
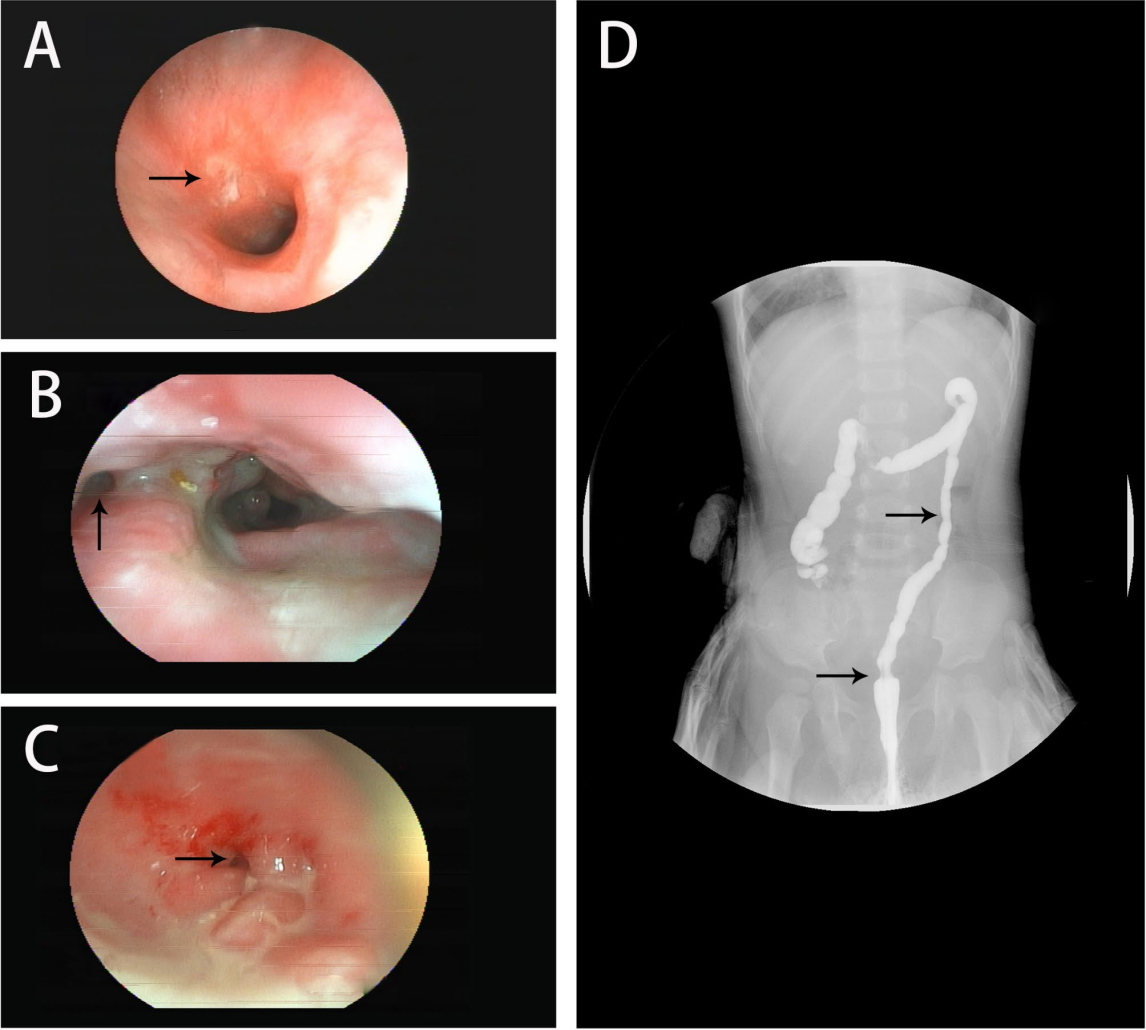
Colonoscopy and radiological picture of patients with IL10R gene mutations. (**A**) anastomotic inflammation and ulcer; (**B**) perianal fissure; rectal fistula; (**C**, **D**). enterostenosis in colonoscopy and barium enema. The arrow head indicates the lesion sites

Of the 25 patients with enterostomy and HSCT, 21 underwent stoma closure; 4 without a stoma were excluded because of the short frame time from HSCT to the draft of this manuscript. The median timeframe between HSCT and stoma closure was 19.6 [15.9,26.2] months. Among the patients who underwent stoma closure, 9 were from the emergency enterostomy group, and 10 were from the selective enterostomy group. The remaining 6 patients were waiting for stoma closure, which was due to perianal lesions in 2 patients with delayed stoma closure time. Of the 6 surviving patients who only underwent enterostomy, 1 was waiting for a suitable match for HSCT, and 5 had no intention of undergoing HSCT for personal reasons (Table 3). Eight patients needed intestine segment resection before the stoma closure was performed (Fig. 2).

**Table 3:** Intestinal/perianal lesions and stoma closure m = month, n = number

Patients underwent stoma closure	
Number of patients	19
Age of stoma closure(m)	44.9 ± 18.5
The duration time of enterostomy (m)	26.2[20.3,32.9]
The interval time between enterostomy and HSCT (m)	19.6[15.9,26.2]
Patients with HSCT + stoma closure (n)	19
Patients with HSCT + without stoma closure (n)	6
Stoma closure patients without HSCT(n)	0
Patients with anoplasty operation (n)	11
Patients with enterolysis (n)	20
Patients with fistulous tract operation (n)	9
Number of patients with partial intestine resection (n)	7

### Risk factor analysis of stoma closure

The surviving patients with enterostomy and HSCT were divided into two groups according to whether the interval time between HSCT and stoma closure was less than 18 months (early stoma closure group, n = 9) or more than 18 months (late closure group, n = 12). The univariate analysis between the early stoma closure and late stoma closure groups is described in Table 4.

**Table 4:** Univariate analysis between normal stoma closure and delayed stoma closure

	Stoma closure (n = 9)	Delay stoma closure^*^ (n = 12)	*P*
Sex (male: female)	4:5	5:7	0.899
Age of diagnosis (m)	11.4 ± 7.5	18.9 ± 12.2	0.122
Age of enterostomy(m)	11.6 ± 6.2	20.1 ± 12.4	***0.081***
Age of HSCT (m)	17.3 ± 8.1	31.3 ± 15.5	***0.023***
BW before HSCT (kg)	8.2 ± 3.2	8.9 ± 4.1	0.707
BMI z score before HSCT	-1.02 ± 2.54	-0.27 ± 1.79	0.435
Enterostomy: emergency vs. selective	8:4	1:8	0.11
SES	24 ± 7	22 ± 8	0.499
Lesions of the anus (n)	3	6	0.445
Fistulous tract (n)	3	6	0.445
Partial intestine stenosis (n)	2	5	0.350

* Two patients with interval time between enterostomy and HSCT more than 18 months without stoma closure were enrolled in delay stoma closure group

Variables with statistical significance in the univariate analysis were considered in the multivariate logistic regression analysis. In a univariate logistic model, risk factors significantly associated with late stoma closure were age at enterostomy and age at HSCT. However, multivariate logistic regression showed no statistically significant factor associated with late stoma closure. No *Clostridium difficile* infections were found in the above two groups, even though infection by this pathogen has been associated with delayed ileostomy closure.

The patients were further followed up after stoma closure. Only one patient had anastomotic inflammation and ulcers and needed subsequent visits. Until the last follow-up for determining the growth and development after stoma closure, there was no significant difference between the stoma closure group and delay closure group in the z scores of WFA (-1.35 ± 1.05 vs.-2.09 ± 1.56, *P* = 0.24), LFA (-2.07 ± 1.20 vs.-3.18 ± 2.31, *P* = 0.21) and BMI (-0.04 ± 1.51 vs.-2.09 ± 1.56, *P* = 0.83).

## Discussion

VEO-IBD represents approximately 25% of cases of IBD-like colitis occurring during childhood and has greater potential for escalated treatment, such as extensive surgery and more intensive medical therapies [9, 10]. The cumulative risk of bowel surgery in children with VEOIBD is approximately 14–15% by 5 years. For severe VEOIBD patients with IL10R gene mutations, enterostomy is always inevitable [11, 12]. Our previous single-center retrospective study reported the complications of enterostomy and related risk factors in 22 VEOIBD patients with IL10R gene signaling deficiency [7]. In this study, we collected 46 IL10R gene-mutated patients with enterostomy and followed up on their outcomes after HSCT, especially influencing factors for stoma closure.

The annual number of patients with enterostomy in our pediatric IBD center increased, as shown in Fig. 1B. There is a tendency for the number to increase because of the attention increasingly given to IL10R gene-mutated monogenic IBD patients. The timely management of VEO-IBD with perianal or severe intestinal lesions is important owing to the possible adverse effects on growth, development, and quality of life [8]. For these patients, a combination of medical and surgical treatment was often required to control diseases [10, 13]. Surgical treatment included emergency enterostomy and selective enterostomy depending on the condition of the disease [14].

In our study, the emergency enterostomy group had a higher number of deaths than in the selective enterostomy group. The reason was that the patients in the emergency enterostomy group had severe illness and complications. Therefore, the patients in our center received medical and surgical intervention, when necessary, regardless of whether they were referred for transplantation [8].

In this study, for IL10R-mutated VEOIBD patients, we found that timely surgery and enterostomy showed benefits for VEOIBD with IL-10 signaling deficiency. The timing of intervention, potential postoperative complications, economic burden and other related problems should be considered when an enterostomy is needed [15]. Surgical intervention should be performed earlier because the perforations in monogenic IBD are usually insidious [16]. Preventative enterostomies are suggested in preparation for HSCT among patients with severe anorectal complications. Clara et al. reported that the absence of perianal/rectal CD activity (HR 3.00; 95% CI 1.86–4.86; *p* < 0.001) emerged as an independent predictor of a shorter time to stoma reversal [17]. However, enterostomy only showed temporary benefits, and increasing time was associated with an increase in complication rates and increased length of hospital stay with loop ileostomy [18]. In our study, only patients with HSCT had the chance for stoma closure. For those patients without HSCT, the enterostomy was still maintained, although they wished for stoma closure.

Åsa et al. reported that stomas were more common in elderly-onset patients than in pediatric-onset patients, with a 5-year cumulative incidence of 3.6% vs. 1.3% [19]. In those patients, ileostomies were most common (64%), and 24.5% of the patients who underwent stoma surgery had perianal disease at the end of follow-up. Within 5 years of diagnosis, 0.8% of the incident patients had a permanent stoma. In our study, we found that 34.6% of the IL10R gene-mutated VEOIBD patients had an enterostomy, and eneterostomies were mainly performed in patients with perianal disease or severe intestinal lesions.

However, stoma closure is needed for the patients with perianal disease or severe intestinal disease to help them improve their quality of life and have good functional outcomes. Tracanelli et al. reported that anti-TNF-α was significantly related to successful closure techniques for patients with a rectovaginal fistula secondary to Crohn’s disease (*p* = 0.007) [20]. Because of the limited number of patients with rare diseases, there was no statistical difference in multiple intestinal lesions at the time of stoma closure. Delayed ileostomy closure was also associated with a nearly 7-fold increase in the risk of *Clostridium difficile* infection (OR = 6.95, CI: 1.06–81.6; *P* = 0.03) [21]. In our study, we did not find any *Clostridium difficile* infections in the delayed stoma closure group. For the VEOIBD patients with mutations in the IL10R gene, we and other groups showed that HSCT was the only possible cure for patients with IL-10 signaling deficiency, although it is associated with complications, including graft failure, GVHD and infections. After HSCT, all patients underwent stoma closure if the timing was appropriate, and the patients had no chance for stoma closure without HSCT. After follow-up, 41.3% (19/46) of our patients had permanent stoma closure.

Vogel, et al. reported a major morbidity rate of 23% (n = 66/292) following stoma closure in young children, most commonly comprising anastomotic leakage/stenosis, incisional hernia and adhesive obstructions [19]. In our study, no patients died after stoma closure, and only anastomotic inflammation and ulcers were found in one patient. Growth and development delay compared with that of peers occurred, even though the patient was much improved.

Our study had limitations. First, VEOIBD with an IL10R gene mutation is still a rare disease, so the number of patients with enterostomy and HSCT was not sufficient, which may lead to statistical bias. For example, the factors of intestinal/perianal lesions and complications of HSCT were not significantly different in the patients with early and late stoma closure group. Future multicenter studies of patients with VEOIBD may be able to distinguish predictors for stoma closure. Furthermore, data on long-term stoma closure are lacking, and more long-term follow-up is needed.

## Conclusions

In summary, this study showed the current status of IL10R gene-mutated VEOIBD patients after enterostomy in China. Prompt and appropriate enterostomy and HSCT may be the best option for improving quality of life in the long term.

## Data Availability

The datasets used and analyzed during the current study are available from the corresponding author on reasonable request.
